# Expansion and Evaluation of Pharmacist Services in Primary Care

**DOI:** 10.3390/pharmacy8030124

**Published:** 2020-07-22

**Authors:** Katherine J. Hartkopf, Kristina M. Heimerl, Kayla M. McGowan, Brian G. Arndt

**Affiliations:** 1 Department of Pharmacy, University of Wisconsin Health, Madison, WI 53792, USA; kheimerl@uwhealth.org (K.M.H.); kmcgowan@uwhealth.org (K.M.M.); 2 Department of Family Medicine and Community Health, School of Medicine and Public Health, University of Wisconsin, Madison, WI 53706, USA; brian.arndt@fammed.wisc.edu; 3 University of Wisconsin Health PATH Collaborative (Primary Care Academics Transforming Healthcare), Madison, WI 53705, USA

**Keywords:** pharmacist, primary care, collaborative practice agreement, patient care extender, comprehensive medication management, quality improvement, electronic health record, electronic dashboard

## Abstract

Challenges with primary care access and overextended providers present opportunities for pharmacists as patient care extenders for chronic disease management. The primary objective was to align primary care pharmacist services with organizational priorities and improve patient clinical outcomes. The secondary objective was to develop a technological strategy for service evaluation. An interdisciplinary workgroup developed primary care pharmacist services focused on improving performance measures and supporting the care team in alignment with ongoing population health initiatives. Pharmacist collaborative practice agreements (CPAs) were developed and implemented. An electronic dashboard was developed to capture service outcome measures. Blood pressure control to <140/90 mmHg was achieved in 74.15% of patients who engaged with primary care pharmacists versus 41.53% of eligible patients electing to follow usual care pathways. Appropriate statin use was higher in patients engaged with primary care pharmacists than in eligible patients electing to follow usual care pathways both for diabetes and ischemic vascular disease (12.4% and 2.2% higher, respectively). Seventeen of 54 possible process and outcome measures were identified and incorporated into an electronic dashboard. Primary care pharmacist services improve hypertension control and statin use. Service outcomes can be measured with discrete data from the electronic health record (EHR), and should align with organizational priorities.

## 1. Introduction

A growing issue in the United States healthcare system is timely access to primary care [1,2,3]. The population is increasingly comprised of aging individuals with a large number of complex and chronic disease states. Effective, long-term management of these chronic disease states requires careful planning and the establishment of attainable health goals [4,5,6]. Additionally, shared decision-making should occur between the patient and provider to ensure that appropriate risks and benefits of care are considered. Furthermore, providers are tasked to ensure patients are equipped with the knowledge they need for active involvement in their own care [7]. Differing patient populations require individualized and often complex approaches to care, which consume significant amounts of healthcare resources, including provider time. 

In many primary care practices, patient care extenders are under-utilized, which leads to unnecessary burden of clinical and nonclinical activities for the provider. Health systems have started to transition responsibility for lab ordering, patient education, medication titration, refills, prior authorizations, and other similar duties to various lower-cost patient care extenders, at times utilizing disease-specific collaborative practice agreements (CPAs) [8,9,10]. Innovative use and delegated expanded roles of patient care extenders in primary care are imperative for improved patient outcomes given the ever-rising demand and time constraints on primary care providers [11,12,13,14]. 

Implementation of patient care extenders in a team-based care model has demonstrated enhanced quality of patient care, improved cost-effectiveness, and reduced provider burnout [15,16,17,18]. Previously published studies suggest pharmacists practicing in primary care can significantly decrease medication errors, improve health outcomes, and enhance provider satisfaction when functioning as integral members of the patient care team [19,20,21,22,23,24]. Measuring and reporting service outcomes in a timely manner is vital to implementation and growth of primary care pharmacist roles and has demonstrated, in some cases, improved evidence-based care and patient health outcomes [25,26]. It also allows for rapid quality improvement and alignment of pharmacist resources based on patient population needs. The necessary data to report service outcomes can and should be leveraged from the electronic health record (EHR) and corresponding data warehouses for optimal efficiency and care team visibility.

## 2. Service Implementation and Evaluation 

### 2.1. Setting

This service was developed at a six-hospital academic medical center (AMC) with 34 primary care clinics and approximately 287,000 medically homed patients. The AMC has residency-focused, community-based, and regional partners in family and internal medicine. Since 2010, the AMC has incorporated efforts to redesign primary care services with specific focus areas of patient-centered care, efficiency, service standardization, and care team member engagement. 

### 2.2. Phase 1 Implementation and Analysis

In 2016, six salaried pharmacist full-time equivalents (FTE) were hired to support further redesign efforts and grow the patient care services offered for comprehensive primary care. Initial pharmacist services were provided to patients with high medication burden (≥13), multiple co-morbid conditions (≥6), and those referred from providers. All initial services were recommendation-based and ultimately required provider approval in order to implement medication changes with patients. As a result, data collection focused on medication interventions completed. Providers were receptive to recommendations from a primary care pharmacist. However, it was challenging to measure value and directly associate pharmacist services with clinical outcomes. Additionally, all data collection was manual, inefficient, and subject to human error. Through partnership with key leaders in the primary care and population health departments at the AMC, the need for pharmacist service revision to better align with organizational population health efforts as well as a dynamic service dashboard was identified. 

### 2.3. Service Revision

The primary objective was to align primary care pharmacist services with organizational priorities and improve patient clinical outcomes. The secondary objective was to develop a direct technological interface with the EHR for pharmacist service evaluation.

A workgroup consisting of the Senior Medical Director of Primary Care, Medical Director of Population Health, Pharmacy Director and Manager of Ambulatory Care Services, and a pharmacy resident convened to redesign primary care pharmacist services. Pharmacist services were developed with information from four sources. Healthcare performance measures, including state-specific quality measures and Accountable Care Organization measures, were assessed to identify opportunities for improvement compared to peer institutions. Proven measures in which pharmacists provide value and improve outcomes were identified using literature searches via PubMed and pharmacy journals. Existing organization-specific primary care workflows and care team roles were considered by the workgroup. Additionally, provider perspectives collected during the intervention-based initial phase of implementation helped inform the service revision proposal.

The top five proposed pharmacy services were identified based on the intersection of organizational need for improvement and published data to support pharmacists improving outcomes in those specific disease states. Additionally, a strategy to expand the existing pharmacist FTE resources to additional primary care clinics and patients was prepared. All aspects of the proposed services aligned with key components of the organization’s population health core standards: clear patient inclusion and exclusion criteria, consistent service aspects during initial and follow-up patient outreach, and clear discharge criteria. The proposal specified the patient care pathway if the provider and patient agreed to participate in the pharmacist service. This pathway included the ability for pharmacist identification or provider referral, patient enrollment processes, visit elements and cadence, and discharge back to primary care provider when clinical goals were met or a patient disengaged. Appropriate patient was defined as a patient that required medication titration, and had a history of medication adherence concerns or numerous adverse effects to medications. This was in contrast to usual care processes in which patients were seen in-clinic for scheduled provider visits. The providers were accountable to determine any necessary changes in medication therapy and schedule appropriate follow-up with a provider, nurse, or medical assistant. The resulting pharmacist service proposal was presented to a primary care leadership committee for feedback and ultimate endorsement.

### 2.4. Phase 2 Implementation

An initial clinic location was identified by the workgroup for implementation of the redesigned pharmacist service. Two primary care pharmacists were assigned to lead the implementation at this location and refine service workflows over a 4-week implementation period. In parallel, CPAs were developed and approved for hypertension and statin medication management to be delegated from providers to trained primary care pharmacists. The structure of the CPAs aligned with organizational requirements and included the following information: patient eligibility criteria, contraindications for use of the CPA, when to consult a provider, treatment goals, documentation requirements, patient follow-up and monitoring, and medications and labs that could be prescribed or ordered by a trained pharmacist. 

A team training occurred at the end of the initial clinic implementation period to share the vision and need for service revisions, as well as train all primary care pharmacist team members on the new workflows. When the CPAs were fully approved for implementation, the pharmacists completed targeted team trainings. Each pharmacist was required to participate in a didactic therapeutic review session with a cardiology specialist physician. Additionally, each pharmacist completed two case-based assessments. The first was for the elements contained in the CPA, and the second was a clinical assessment. Pharmacists were required to receive a score of 80% in order to utilize the CPA. In addition to CPA training, all pharmacists pursued board certification in ambulatory care within their first year of hire.

Utilizing a standardized clinic implementation toolkit, the redesigned pharmacist services were implemented at an average of one clinic per month over the span of one year. The clinic implementation toolkit assisted each pharmacist in integrating services into the clinic. Pharmacists were responsible for setting up their patient room, ongoing communication with staff, and sharing successes and barriers with building their patient panels. The EHR integration with clinics included creating a pharmacist schedule, clinic communication pool or in-basket, and pharmacist referral that could be entered by providers.

Continued workgroup meetings identified core process and outcome measures to be tracked and reported to demonstrate service success. A prioritization matrix, a quality improvement tool, was used with the analytics team to identify effort to access and impact of the proposed outcome measures [27,28]. Through engagement and collaboration with analytics and information technology representatives, a service-specific dashboard was developed in external software used throughout the organization. Additionally, a new electronic form was developed within the EHR to capture service process measures in a discrete manner and inform the dashboard. The electronic form was completed by the pharmacist for every patient the pharmacist intended to enroll in services. While the form was open, the pharmacist was providing services to enrolled patients and considered them as part of their panel. Then, upon discharge, the form was completed in its entirety.

This project was determined not to meet the federal definition of research, and the UW-Madison Health Sciences Institutional Review Board certified it as a quality improvement project.

## 3. Results

The AMC’s primary care pharmacist service was redesigned to focus on core services linked to specific clinical outcomes in alignment with selected healthcare performance measure definitions. Hypertension medication management to achieve goal blood pressure <140/90 mmHg, and statin initiation for patients with diabetes or ischemic vascular disease, was completed via CPA to offset provider workload. Individualized patient goals and medication management recommendations presented to patients included assessment of perceived benefits and risks to promote shared decision-making. Pharmacists also remained available for comprehensive medication reviews and care team clinical consults due to provider demand for assisting in care management of complex patients. These services were not directly related to the core services, as they were not linked to specific clinical outcomes. Pharmacists were onsite one to three days per week across 13 of the 34 primary care clinics. When not onsite, services were supported by a centralized triage pharmacist shift. 

The CPAs for hypertension and statin services were initiated by provider referral or pharmacist identification via disease state registries. Within the first year of the service revision, 948 patients were identified as eligible for the hypertension service, with 607 (64%) patients engaging in at least one clinical visit with a primary care pharmacist. Clinical measures demonstrated blood pressure was controlled to <140/90 mmHg in 74.15% of patients who engaged with a primary care pharmacist versus 41.53% of eligible patients electing to follow usual care pathways, a difference of 32.6% (Appendix A). 

Statin use when indicated was also higher when patients engaged with a primary care pharmacist for an assessment of statin appropriateness, discussed risks versus benefits, and decided whether or not to pursue statin therapy. In the first year of the service revision, 481 patients with diabetes and not on a statin engaged with a primary care pharmacist versus 243 eligible patients who did not engage with the pharmacist. Statin use when indicated was 12.4% higher in the group of patients engaging with the pharmacist service than in those following usual care pathways (82.93% and 70.56%, respectively) (Appendix B). There was also a modest increase in statin use of 2.2% for patients with ischemic vascular disease (98.21% with pharmacists and 96% with usual care) (Appendix C). 

Based on a conservative estimate of the lifetime cumulative costs of one fatal cardiovascular event, the first year of the revised services for hypertension management and statin initiation alone produced greater than $1.4 million in cost avoidance [29,30,31,32]. 

In order to develop ongoing service evaluation monitoring, 54 proposed process and outcome measures were evaluated using a prioritization matrix (Appendix D). Seventeen measures were identified with the highest feasibility and greatest impact on service success, and informed the data collection plan. The electronic form developed within the EHR directly linked service patients through an episode of care and discretely tracked the identified process metrics through eight targeted questions. The service-specific dashboard updated daily to provide dynamic information. The dashboard was designed to house individual reports and information that aligned with the data collection plan. Two reports displayed results of the clinical outcomes of service patients and tracked the results over time. Five additional reports linked directly to patient-level data to allow for targeted assessment of outliers and identification of service improvement opportunities. Additionally, two reports emphasized areas of interest from a population health perspective: demographics and mix of patients from our accountable population.

## 4. Discussion

The medication management responsibilities within this primary care pharmacist service align with the American College of Clinical Pharmacy (ACCP) definition of clinical pharmacists who provide direct patient care [33]. The pharmacists engage in direct assessment and evaluation of patient-specific medication needs; medication selection, initiation, titration, and discontinuation; ongoing monitoring of lab results, side effects, and adherence; and necessary communication with providers when concerns arise. Pharmacists are well trained and prepared to provide this level of patient care, especially in team-based care models [34].

Though this was not a robust, randomized trial of clinical outcomes, this primary care pharmacist service further endorses the positive impact of a pharmacist embedded in the care team. When serving as a patient care extender, the optimal role for a pharmacist involves medication management for chronic disease states. These activities transition workload from providers, and can be directly associated with improving measurable patient health outcomes. As health systems transition to value-based payment models, pharmacists can positively impact quality measures for chronic disease states where medication optimization is key for disease control and prevention of poor outcomes, such as cardiovascular disease [35,36,37,38]. 

There are several limitations of our service evaluation. One notable limitation is patient self-selection bias. Patients who engaged with a pharmacist may be more motivated to improve their health outcomes. Alternatively, patients referred to a pharmacist for medication management may be more complex than patients seen by a nurse or medical assistant through usual care. Randomization would have reduced potential bias; however, it was not pursued due to the quality improvement nature of this work. Another limitation is that several patients followed usual care pathways instead of working with a pharmacist because of capacity. The six pharmacist FTE are not able to reach all patients, so targeted efforts and patient prioritization is key. 

An additional key limitation of our year one evaluation and dashboard development has been an inability to capture the primary care pharmacist service impact of comprehensive medication reviews for patients at risk of readmission. This, in addition to service impact on unintended hospitalizations, will be considered for future service evaluation.

While healthcare performance measures are beneficial to benchmark success, they can be restrictive and may not fully reflect the service impact. Given the inclusion of shared decision-making between pharmacist and patient, there are patients who believe the possible benefit does not outweigh the potential risk and the resulting outcome does not positively align with the designated performance measure. 

The service redesign efforts and processes help guide others through a process of evaluating organizational need and population health standards to align services provided by pharmacists in primary care. It also demonstrates identification of a systematic data collection plan that will interface with discrete information available through the EHR to inform a dashboard with meaningful, dynamic, and timely data. All process and clinical results provided in this year-one evaluation were extracted directly from the service dashboard, required no manipulation, and continue to be updated daily. The data can be utilized for ongoing quality improvement and to support realignment and growth of services to meet changing organizational needs in alignment with prior research [39,40].

## 5. Conclusions

There is an opportunity in the primary care setting to introduce new clinical pharmacist services, along with an effort to reallocate medication management activities from providers, while aligning with organizational priorities such as improved hypertension control and statin use. Clear definition of pharmacist service measures and a direct interface with discrete data from the EHR allows for optimal evaluation of both clinical and operational impacts.

## Appendix A

**Table A1:** Blood Pressure Control.

Blood Pressure Control (n = 948)				
Year	Month	% Controlled RPh (n = 607)	% Controlled Non-RPh (n = 341)	(% Controlled RPh)– (% Controlled Non-RPh)
2017	Jun	70.7	60.66	10.0
	Jul	71.67	59.78	11.9
	Aug	67.04	58.60	8.4
	Sept	67.13	54.23	12.9
	Oct	35.78	52.36	13.4
	Nov	63.61	50.53	13.1
	Dec	61.04	44.16	16.9
2018	Jan	63.59	44.39	19.2
	Feb	63.43	42.59	20.8
	Mar	64.06	43.66	20.2
	Apr	66.3	40.08	26.2
	May	70.41	40.98	29.4
	Jun	74.15	41.53	32.6
	Jul	73.6	41.04	32.6
	Aug	73.98	43.49	30.8

## Appendix B

**Table A2:** Statin Use—Diabetes.

Statin Use in Patients with Diabetes (n = 724)				
Year	Month	% Statins RPh (n = 481)	% Statins Non-RPh (n = 243)	(% Statins RPh)–(% Statins Non-RPh)
2017	Jun	71.3	59.75	11.6
	Jul	72.31	59.26	13.1
	Aug	72.36	58.18	14.2
	Sept	72.81	57.99	14.8
	Oct	71.94	59.77	12.2
	Nov	73.98	61.33	12.7
	Dec	75.86	62.64	13.2
2018	Jan	76.86	63.89	13.0
	Feb	77.27	64.25	13.0
	Mar	78.75	66.84	11.9
	Apr	79.83	66.56	13.2
	May	81.74	68.72	13.0
	Jun	82.93	70.56	12.4
	Jul	83.02	69.90	13.1
	Aug	83.24	70	13.2

## Appendix C

**Table A3:** Statin Use—Ischemic Vascular Disease.

Statin Use in Patients with IVD (n = 278)				
Year	Month	% Statins RPh (n = 195)	% Statins Non-RPh (n = 83)	(% Statins RPh)–(% Statins Non-RPh)
2017	Jun	93.26	86.05	7.2
	Jul	93.41	84.09	9.3
	Aug	93.41	84.09	9.3
	Sept	93.41	85.71	7.7
	Oct	93.62	88.37	5.3
	Nov	94.62	90.91	3.7
	Dec	95.83	89.58	6.3
2018	Jan	95.05	89.8	5.3
	Feb	96.04	89.58	6.5
	Mar	96.15	93.33	2.8
	Apr	96.49	93.48	3.0
	May	97.32	93.75	3.6
	Jun	98.21	96	2.2
	Jul	99.07	96	3.1
	Aug	99.12	96.23	2.9

## Appendix D

**Table A4:** Measure Prioritization.

Priority	Measure
5–11	Wisconsin Collaborative for Healthcare Quality Outcomes a. Hypertension: Controlling high blood pressure b. Hypertension: Daily aspirin or other antiplatelet for diabetes patients c. Diabetes: Statin use d. Diabetes: Daily aspirin or other antiplatelet for diabetes patients e. Ischemic vascular disease (IVD): Blood pressure control f. Ischemic vascular disease (IVD): Daily aspirin or other antiplatelet unless contraindicated g. Ischemic vascular disease (IVD): Statin use
1–4	Service Success a. Patient dropout rate b. Reason for service graduation/discontinuation c. Length of time spent in service d. Number of patients enrolled
13–14	Readmission a. Readmission reduction (30 and 90 day) b. Cost avoidance of potential readmissions
16–17	Satisfaction Patient satisfaction with pharmacist services Provider satisfaction with pharmacist services
12	Patients per pharmacist per hour
15	Adherence to the CPAs for hypertension and lipids
